# Editorial: Xenobiotics from diet and health: impact on microbiome

**DOI:** 10.3389/fnut.2023.1342142

**Published:** 2024-01-04

**Authors:** David Ríos-Covian, Maria Carmen Collado, Carina Venter, Carlos Gómez-Gallego, Clara G. de los Reyes-Gavilán, Sonia González

**Affiliations:** ^1^Equipe Interactions des Micro-organismes Commensaux et Probiotiques avec l'Hôte, Institute MICALIS, National Research Institute for Agriculture, Food and the Environment, Univ Paris Saclay, Paris, France; ^2^Department of Biotechnology, Institute of Agrochemistry and Food Technology-National Research Council, Valencia, Spain; ^3^Section of Allergy and Immunology, Department of Pediatrics, Children's Hospital Colorado, University of Colorado School of Medicine, Aurora, CO, United States; ^4^Department of Pediatrics, Institute of Public Health and Clinical Nutrition, University of Eastern Finland, Kuopio, Finland; ^5^Department of Microbiology and Biochemistry of Dairy Products, Instituto de Productos Lácteos de Asturias (IPLA-Spanish National Research Council), Villaviciosa, Spain; ^6^Diet, Microbiota, and Health Group, Instituto de Investigación Sanitaria del Principado de Asturias (DIMISA, Health Research Institute of Asturias), Oviedo, Spain; ^7^Department of Functional Biology, Faculty of Medicine, University of Oviedo, Oviedo, Spain

**Keywords:** xenobiotics, detoxicant, probiotics, *Lactobacillus*, nitrosocompounds, macromolecular polysaccharide

## Introduction

This Research Topic collects studies that assess the impact of different dietary components on the composition of the gut microbiota. Although *sensu stricto* the term “xenobiotic” refers to a chemical substance found in an organism that is not naturally produced or expected to be present in that organism, it is also commonly applied to substances with possible toxic or carcinogenic action, such as environmental pollutants or pesticides (1). It has been estimated that humans are exposed to 1–3 million xenobiotics during their lifetime, so this plethora of compounds makes this an attractive and emerging field of study (1).

The majority of the substances that fall under the term xenobiotic have become relevant because of the changes in our dietary patterns over the past few years, with a remarkable increase in food processing, but also as a result of the application of certain techniques that enhance the palatability of foodstuffs. This is the case of heterocyclic amines (HA), polycyclic aromatic hydrocarbons (PAH), acrylamides generated by cooking food at high temperatures, or glycation end products (AGEs) (2–4). The latter are formed by non-enzymatic reactions between carbonyl groups of reducing sugars and free amino groups in foods cooked at high temperatures or stored for long periods (5). The increase in the concentration of these compounds in the body is directly associated with the degree of inflammation, the formation of free radicals, insulin resistance, and metabolic disturbances (4). In this regard, the article by Park et al. evaluates the impact of a strain of *Lactococcus lactis* isolated from kimchi (LL-KF140) on the toxicokinetics of Nε-(carboxymethyl)lysine (CML), one of the most abundant glycosylation end products in foods. The paper includes data from an *in vitro* assay to test the efficacy of eight enzymes produced by strain LL-KF140 in reducing CML after 24 h of incubation, an *in vivo* study in a rat model treated with CML as a casein-lactose reaction product, and the bacteria administered for 14 d. These researchers also carried out a clinical trial in humans, who received the bacteria for 26 d together with CML administered in 40 g of parmesan cheese. The results presented in this work contribute to expanding the still-limited knowledge about the role of Lactobacilli as detoxifiers of compounds that may be harmful to human health.

Similarly, Zapico et al. have evaluated the impact of exposure to various food cooking by-products in the diet of a sample of socially vulnerable individuals on the composition and activity of the gut microbiota. The regular diet of the subjects leads to the intake of a variety of heterocyclic amines, nitrates, nitrites, nitrosocompounds, polycyclic aromatic hydrocarbons, and acrylamide. A comparison of xenobiotic intake with fecal microbiota composition revealed that the Pseudomonadota and Verrucomicrobiota phyla showed significant correlations with the intake of some xenobiotics (heterocyclic amines, polycyclic aromatic hydrocarbons, and nitrates). In addition, MelQx had a negative association with the microbial families *Lachnospiraceae* and *Eggerthellaceae*, while PhIP was related to the relative abundance of the *Muribaculceae, Streptococcaceae*, and *Eubacterium coprostanoligenes* groups. These results support the possible association between xenobiotics derived from food processing and gut microbiota composition.

The work of Liu et al. addresses another important aspect of this complex relationship between diet and microbiota. Several studies in recent years have found differences in the composition of the microbiota according to gender (6). However, these researchers go a step further by studying the different responses in terms of microbiota composition after the administration of the macromolecular polysaccharide *Inonotus obliquus polysaccharide* (IOP) in male and female rats (Liu et al.). For this purpose, they determined the molecular weight and purity of IOP by high-performance gel permeation chromatography (HPGPC) and the phenol sulfuric acid method, with NMR used to confirm the chemical structure of IOP. Sex hormone [testosterone (T) and estradiol (E2)] levels and intestinal microbial changes were detected by enzyme-linked immunosorbent assay (ELISA) and 16S rRNA, respectively, after IOP (100 mg/kg) gavage in male and female Sprague-Dawley (SD) rats. It was observed that IOP induced apoptosis in a dose-dependent manner, with 46.19% of apoptosis occurring at high doses. In the rat model, the administration of IOP was associated with an increase in the levels of the hormone E2 in male rats with respect to the control. IOP was associated with the increased abundance of *Lactobacillus, Roseburia*, and *Clostridia*_UGC-014 in female rats, whereas in male rats, the genera *Prevotella, Alistipes*, and *Clostridia*_UGC-014 were increased. Thus, this study supports the existing knowledge of the IOP structure and elucidates the modifications that occur in the intestinal microbiota following IOP administration in rats of both sexes.

The present Research Topic and special edition are concluded with a review by Muratore et al. on nutritional modulation of the microbiome in recipients of allogeneic hematopoietic stem cell transplantation. Allogeneic hematopoietic stem cell transplantation (allo-HSCT), used as a treatment for various oncologic and non-oncologic diseases, is associated with high morbidity and mortality. In this work, the authors propose that intestinal dysbiosis may lead to complications that could increase the risk of mortality. This reinforces the interest in identifying different nutritional strategies to improve the quality of life in cancer patients and reduce the risk of mortality through modulation of the intestinal microbiota. In this article, a complete overview of how the pre-transplant diet could affect the composition of the microbiome and its ability to resist the alterations that occur during transplantation is provided. This emphasizes the importance of enteral or parenteral nutrition (7, 8).

## Conclusion

The results reported in this Research Topic, together with those that will be developed from them, will allow the discovery of different compounds with the ability to modulate the intestinal microbiota and decipher the mechanisms that explain this interaction. This will be the first step toward the development of new dietary strategies capable of counteracting the potential adverse effects that these compounds may have. This compendium of work highlights the importance of future assessment of the relationship between xenobiotic intake and microbiota composition, as recently reported by the European Food Safety Agency (9).

## Author contributions

DR-C: Writing – review & editing. MC: Writing – review & editing. CV: Writing – review & editing. CG-G: Writing – review & editing. CR-G: Conceptualization, Writing – review & editing. SG: Conceptualization, Writing – original draft, Writing – review & editing.
